# Prognostic implications of EGFR protein expression in sporadic colorectal tumors: Correlation with copy number status, mRNA levels and miRNA regulation

**DOI:** 10.1038/s41598-020-61688-7

**Published:** 2020-03-13

**Authors:** Sofía del Carmen, Luís Antonio Corchete, Ruth Gervas, Alba Rodriguez, María Garcia, José Antonio Álcazar, Jacinto García, Oscar Bengoechea, Luis Muñoz-Bellvis, José María Sayagués, Mar Abad

**Affiliations:** 1 Department of Pathology and IBSAL, University Hospital of Salamanca, Salamanca, Spain; 2 Cancer Research Center and Hematology Service (University Hospital of Salamanca), Salamanca, Spain; 3 Department of Oncology, University Hospital of Salamanca, Salamanca, Spain; 4 General and Gastrointestinal Surgery Service, University Hospital of Salamanca, Salamanca, Spain

**Keywords:** Cancer genomics, Diagnostic markers

## Abstract

Sporadic colorectal cancer (sCRC) is the third most frequent cancer worldwide and the second most common cause of cancer-related deaths (mainly due metastatic dissemination). We investigated the immunohistochemical expression of frequently altered proteins in primary tumors from 51 patients (25 liver metastatic and 26 non-metastatic cases) with a median 103 months follow-up (103 months). We evaluated *EGFR* copy number (using SNP arrays and FISH) and its expression and regulation (by mRNA and miRNA arrays). We found differences between metastatic and non-metastatic sCRCs for MLH1 (*p* = 0.05), PMS2 (*p* = 0.02), CEA (*p* < 0.001) and EGFR (*p* < 0.001) expression. EGFR expression was associated with lymph node metastases (*p* = 0.001), liver metastases at diagnosis (*p* < 0.001), and advanced stage (*p* < 0.001). There were associations between EGFR expression-, *EGFR* gene copy number- and EGFR mRNA levels. We found potential interactions of two miRNAs targeting EGFR expression, (miR-134 and miR-4328, in non-metastatic and metastatic tumors, respectively). EGFR expression was associated with a worse outcome (*p* = 0.005). Multivariate analysis of prognostic factors for overall survival identified that, the expression of EGFR expression (*p* = 0.047) and pTNM stage (*p* < 0.001) predicted an adverse outcome. *EGFR* expression could be regulated by amplification or polysomies (in metastatic tumors), or miRNAs (miRNA-134, in non-metastatic tumors). EGFR expression in sCRC appears to be related to metastases and poor outcome.

## Introduction

Sporadic colorectal cancer (sCRC) is the third most common cancer diagnosed worldwide and the second most frequent cause of cancer-related mortality^1,2^. Around 300,000 new cases of sCRC are reported annually and approximately 67% of these patients die from complications related to sCRC. Metastatic dissemination of the primary tumor, mostly liver metastasis, is the main cause of death of sCRC patients^3^. It is widely believed that the genetic and biological markers associated with the ability of sCRC to invade distant tissues are already present within the primary tumor cells^4,5^. Determining the markers that would help identify patients at risk of harboring or developing metastases could significantly facilitate development of new strategies for diagnosingand managing the disease.

We and others researchers have demonstrated that metastatic sCRC-specific genomic alterations, e.g., del(17p) and del(22q), are common to primary tumors and their paired liver metastatic samples^5–8^, but are absent from non-metastatic sCRC tumors6. Notably, such genomic alterations of sCRC are strongly associated with unique gene expression profiles (GEPs)^9^.

Despite the exceptional utility of genomic methods in the discovery phase of experimentation, their use is limited in most hospitals because they are expensive techniques and difficult to apply in paraffin-embedded material. For these reasons, in practice, most routine diagnoses are performed using immunohistochemistry (IHC) techniques. These drawbacks hinder the clinical implementation of novel biomarkers of disease. In this context, IHC is highly valuable for biomarker validation for several reasons: (i) it enables biomarker expression to be directly visualized in histologically important regions of the tissue; (ii) clinical laboratories usually carry out IHC on formalin-fixed, paraffin-embedded tissue sections that have been processed by standard methods, potentially yielding hundreds of millions of specimens for study^10^; and (iii) validated IHC assays may be incorporated with ease into clinical practice.

Nevertheless, the development of miRNA technology and deep-sequencing techniques has provided tools for the detection and discovery of regulatory RNA molecules^11^. Several miRNA registry databases (miRDB v21, TargetScan 7.2, miRWalk 3, and miRTarBase 7.0) feature details of more than 2000 human miRNA genes, of which 134 were predicted to be potential regulators of EGFR expression. In this study, we searched the miRDB v21 database (http://www.mirdb.org) for candidate miRNAs that regulate EGFR expression, and examined whether the predicted miRNA regulators of EGFR displayed expression levels that were inversely correlated with expression levels of the EGFR gene. We identified EGFR as a potential target of miRNA-134 and miRNA-4328, for non-metastatic and metastatic tumors, respectively.

In the present study, we used IHC techniques to investigate the prognostic value of the expression of the proteins most commonly altered in the primary colorectal carcinomas of 51 sCRC patients (25 liver metastatic and 26 non-metastatic cases) with a long median follow-up. We also analyzed the expression and regulation of the EGFR gene by mRNA and miRNA genes using high-throughput arrays. Copy number alteration (CNA) of the EGFR gene was examined using 500 K SNP arrays and FISH techniques. Overall, our study revealed that EGFR protein expression and copy number are closely related and that EGFR expression, as shown by IHC, is an independent prognostic factor of overall survival (OS) in sCRC patients.

## Results

### Immunohistochemistry

All tumors were positive for CK20 and CDX2, confirming their enteric origin. For most primary antibodies analyzed, sCRC with liver metastases and non-metastatic tumors had similar expression profiles, including similar (*p* > 0.05) levels of expression of MSH2, MSH6, c-Myc, Her2, p53, β-catenin, and Ki-67 antibodies (Table 1). The only statistically significant differences identified between liver metastatic and non-metastatic sCRCs were those involving antibodies MLH1 (*p* = 0.05), PMS2 (*p* = 0.02), CEA (*p* < 0.001) and EGFR (*p* < 0.001). All cases showing loss of MLH1 (20% of cases) and PMS2 (25%) expression, and absence of CEA expression (22% of cases) were non-metastatic tumors; most liver metastatic tumors had a positive value of EGFR membranous expression (79% *vs*. 38% of non-metastatic cases). It should be noted that none of the non-metastatic tumors showed ≥35% positivity for EGFR antibody (Table 1).

**Table 1:** Immunohistochemical expression in patients with metastatic (n = 25) and non-metastatic (n = 26) sporadic colorectal cancer (sCRC) at diagnosis.

Marker	Non-metastatic sCRC (n = 26)	Metastatic sCRC (n = 25)	*p*	Total (n = 51)
**MLH1**				
Loss	5 (20%)	0 (0%)	0.050	5 (10%)
Normal	20 (80%)	25 (100%)		45 (90%)
Excluded	1	0		1
**PMS2**				
Loss	6 (25%)	0 (0%)	0.020	6 (14%)
Normal	18 (75%)	18 (100%)		36 (86%)
Excluded	2	7		9
**MSH2**				
Loss	2 (8%)	0 (0%)	NS	2 (4%)
Normal	22 (92%)	23 (100%)		45 (96%)
Excluded	1	2		3
**MSH6**				
Loss	2 (8%)	0 (0%)	NS	2 (4%)
Normal	23 (92%)	24 (100%)		47 (96%)
Excluded	1	1		2
**c-Myc**				
<10%	2 (8%)	7 (29%)	NS	9 (19%)
≥10%	22 (92%)	17 (71%)		39 (81%)
Excluded	2	1		3
**Her2**				
0/1+	26 (100%)	23 (96%)	NS	49 (98%)
2+	0 (0%)	0 (0%)		0 (0%)
3+	0 (0%)	1 (4%)		1 (2%)
Excluded	0	1		1
**p53**				
0	6 (23%)	7 (29%)	NS	13 (26%)
<10%	9 (35%)	6 (35%)		15 (30%)
≥10%	11(42%)	11 (46%)		22 (44%)
Excluded	0	1		1
**β-catenin**				
Negative	18 (69%)	11 (46%)	NS	29 (58%)
Positive	8 (31%)	13 (54%)		21 (42%)
Excluded	0	1		1
**CEA**				
Negative	11 (22%)	0 (0%)	<0.001	11 (22%)
Positive	15 (88%)	24 (100%)		39 (78%)
**EGFR**				
0	16 (62%)	5 (21%)	<0.001	21 (42%)
1–10%	4 (15%)	1 (4%)		5 (10%)
10–35%	6 (23%)	4 (17%)		10 (20%)
>35%	0	14 (58%)		14 (28%)
Excluded	0	1		1
**Ki-67**				
<50%	15 (58%)	19 (83%)	NS	34 (69%)
≥50%	11 (42%)	4 (17%)		15 (31%)
Excluded	0	2		2

Results expressed as number of cases (percentage); CEA: carcinoembryogenic antigen; NS: no statistically significant differences found between groups (p > 0.05). Cases excluded due to technical problems were not considered in the statistical analysis.

### Association between the immunohistochemical profile of sCRC tumors and other features of the disease

Expression of EGFR > 35% was associated with the presence of lymph node metastases (*p* = 0.001), liver metastases at diagnosis (*p* < 0.001), advanced stages of the disease (*p* < 0.001) and *KRAS* mutations (*p* = 0.001). In contrast, β-catenin expression was significantly associated with microsatellite instability (*p* = 0.007) and *NRAS* mutations (*p* = 0.01). In turn, patients who displayed moderate or intense positivity for CEA antibody were more frequently associated with localization in the rectum (*p* = 0.03), advanced stages (stage IV; *p* = 0.007), presence of lymph node metastases (*p* = 0.01), liver metastases (*p* = 0.003) and *BRAF* mutations (*p* = 0.008). As shown in Table 2, c-Myc expression was significantly associated with advanced stages (stage IV; *p* = 0.03), while patients showing Ki-67 expression were significantly more likely to exhibit microsatellite instability (*p* = 0.03) and BRAF mutation (*p* = 0.04) than those without such expression.

**Table 2:** Expression of EGFR, β-catenin, CEA, c-Myc and Ki-67 antibodies and their association with other clinical, biological, histopathological and genetic features of sporadic colorectal cancer patients (n = 51).

Variable	EGFR		*p*	β-catenin		*p*	CEA		*p*	c-Myc		*p*	Ki-67		*p*
	<35%	≥35%		−	+		−	+		<10%	≥10%		<50%	≥50%	
**Age (years)**															
<72	21 (78)	6 (22)	NS	15 (55)	12 (45)	NS	5 (19)	22 (81)	NS	6 (22)	21 (78)	NS	20 (74)	7 (26)	NS
≥72	15 (65)	8 (35)		14 (61)	9 (39)		6 (26)	17 (74)		3 (17)	18 (83)		15 (65)	8 (35)	
**Gender**															
Male	25 (68)	12 (32)	NS	23 (62)	14 (38)	NS	9 (24)	28 (76)	NS	7 (19)	29 (81)	NS	24 (65)	13(35)	NS
Female	11 (85)	2 (15)		6 (46)	7 (54)		2 (15)	11 (85)		2 (17)	10 (83)		11 (85)	2(15)	
**Site of primary tumor**															
Colon	28 (76)	9 (24)	NS	22 (59)	15 (41)	NS	11 (30)	26 (70)	**0.030**	5 (14)	31 (86)	NS	25 (68)	12 (32)	NS
Rectum	8 (62)	5 (38)		7 (46)	6 (54)		0 (0)	13 (100)		4 (33)	8 (67)		10 (77)	3 (23)	
**Histopathological grade**															
Well differentiated	24 (71)	10 (39)		20 (59)	14 (41)		7 (21)	27 (79)		7 (21)	26 (79)		21 (62)	13 (38)	NS
Moderately differentiated	11 (79)	3 (21)	NS	8 (57)	6 (43)	NS	4 (29)	10 (71)	NS	2 (14)	12 (86)	NS	12 (86)	2 (14)	
Poorly differentiated	1 (50)	1 (50)		1 (50)	1 (50)		0 (0)	2 (100)		0 (0)	1 (100)		2 (100)	0 (0)	
**pT stage**															
T1-T2	8 (100)	0	NS	3 (38)	5 (62)	NS	3 (38)	5 (62)	**0.007**	0 (0)	8 (100)	NS	5 (62)	3 (38)	NS
T3-T4	28 (67)	14 (33)		16 (38)	26 (62)		8 (19)	34 (81)		9 (22)	31 (88)		30 (71)	12 (29)	
**Lymph node involvement**															
N0	30 (86)	5 (14)	**0.001**	21 (62)	13 (38)	NS	11 (44)	24 (56)	**0.010**	5 (15)	28 (85)	NS	24 (69)	11 (31)	NS
N1/N2	6 (40)	9 (60)		8 (50)	8 (50)		0 (0)	15 (100)		4 (27)	11 (73)		11 (73)	4 (27)	
**Liver metastases at diagnosis**															
No	29 (94)	2 (6)	**<0.001**	19 (63)	11 (37)	NS	11 (35)	20 (65)	**0.003**	3 (10)	26 (90)	NS	19 (61)	12 (39)	NS
Yes	7 (37)	12 (63)		10 (50)	10 (50)		0 (0)	19 (100)		6 (32)	13 (68)		6 (31)	13 (69)	
**pTNM stage at diagnosis**															
I	7 (100)	0		2 (29)	5 (71)		3 (43)	4 (57)		0 (0)	7 (100)		4 (57)	3 (43)	NS
II	19 (95)	1 (5)	**<0.001**	15 (79)	4 (21)	NS	6 (30)	14 (70)	**0.007**	2 (11)	16 (89)	**0.030**	12 (30)	18 (70)	
III	3 (75)	1 (25)		2 (50)	2 (50)		2 (50)	2 (50)		1 (25)	3(75)		3 (75)	1 (25)	
IV	7 (37)	12 (63)		10 (50)	10 (50)		0 (0)	19 (100)		6 (31)	13 (69)		16 (84)	3 (16)	
**Tumor size**															
<4 cm	8 (80)	2 (20)	NS	5 (50)	5 (50)	NS	0 (0)	10 (100)	NS	2 (20)	8 (80)	NS	9 (90)	1 (10)	NS
≥4 cm	28 (70)	12 (30)		24 (60)	16 (40)		11 (28)	29 (72)		7 (18)	31 (82)		26 (65)	14 (35)	
**Microsatellite instability**															
No	27 (66)	14 (34)	NS	20 (49)	21 (51)	**0.007**	4 (10)	37 (90)	**<0.001**	9 (23)	30 (77)	NS	32 (78)	9 (22)	**0.030**
Yes	8 (100)	0		8 (100)	0 (0)		8 (100)	0 (0)		0 (0)	8 (100)		3 (37)	5 (63)	
***KRAS*** **mutation**															
No	35 (78)	10 (22)	**0.001**	26 (58)	19 (42)	NS	11 (24)	34 (76)	NS	7 (16)	36 (84)	NS	31 (69)	14 (31)	NS
Yes	0	4 (100)		3 (75)	1 (25)		0 (0)	4 (100)		2 (50)	2 (50)		3 (75)	1 (25)	
***NRAS*** **mutation**															
No	33 (75)	11 (25)	NS	18 (53)	16 (47)	**0.010**	10 (23)	34 (77)	NS	8 (19)	34 (81)	NS	30 (68)	14 (32)	NS
Yes	2 (50)	2 (50)		0 (0)	4 (100)		1 (25)	3 (75)		0 (0)	4 (100)		3 (75)	1 (25)	
***BRAF*** **mutation**															
No	31 (89)	4 (11)	NS	26 (58)	19 (42)	NS	8 (18)	37 (82)	**0.008**	8 (20)	31 (80)	NS	33 (73)	12 (28)	**0.040**
Yes	4 (100)	0		1 (25)	3 (75)		3 (75)	1 (25)		1 (25)	3 (75)		1 (25)	3 (75)	

Results expressed as number of cases (percentage); CEA: carcinoembryogenic antigen; NS: no statistically significant differences found between groups (p > 0.05).

### Impact of immunohistochemical profile on patient overall survival

**Figure 1:**
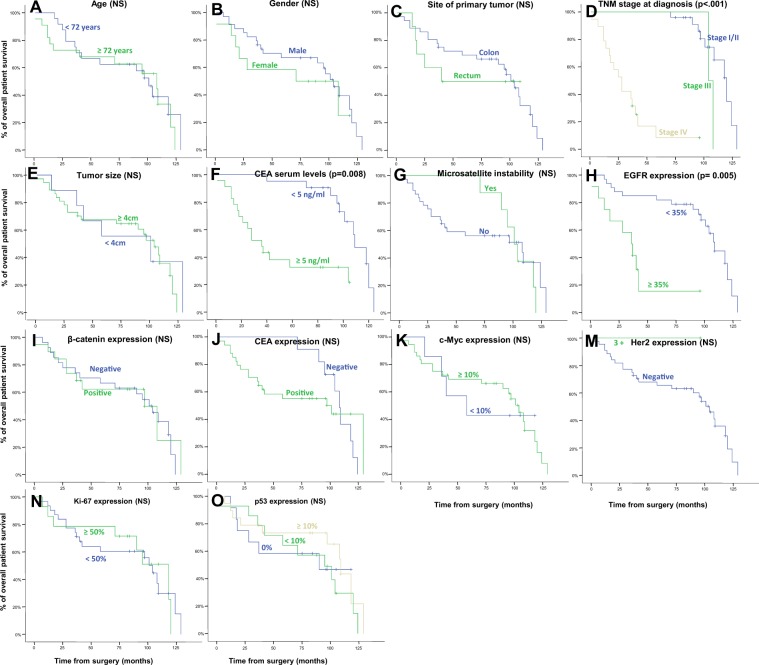
Clinical, biological and immunohistochemical markers of sCRC patients showing the impact on overall survival in the univariate analysis: (**A**) Age, (**B**) gender, (**C**) site of primary tumor, (**D**) TNM stage at diagnosis, (**E**) tumor size, (**F**) carcinoembryonic antigen (CEA) serum levels, (**G**) microsatellite instability, (**H**) EGFR expression, (**I**) β-catenin expression, (**J**) CEA expression, (**K**) c-Myc expression, (**M**) Her2 expression, (**N**) Ki-67 expression, and (**O**) p53 expression.

EGFR expression was associated with a significantly worse outcome (*p* = 0.005; Fig. 1 and Table 3). Advanced TNM stage (*p* < 0.001) and CEA serum levels greater than 5 ng/ml (*p* = 0.008) were also associated with an adverse impact on patient OS (Fig. 1).

**Table 3:** Clinical, biological and immunohistochemical characteristics of sporadic colorectal cancer patients (n = 50) and their association with overall survival.

	N	Univariate analysis		Multivariate analysis	
		HR (95% CI)	*p*-value	HR (95% CI)	*p*-value
**Variable**					
Age					
<72 years	28 (55%)	Ref.	0.7		
≥72 years	23 (45%)	1.14 (0.56–2.31)			
Gender					
Male	38 (76%)	Ref.	0.9		
Female	13 (25%)	0.94 (0.40–2.18)			
Site of primary tumor					
Colon	37 (73%)	Ref.	0.8		
Rectum	14 (27%)	1.13 (0.48–2.66)			
TNM stage at diagnosis					
StageI/II	27 (53%)	Ref.	<0.001*	Ref.	
Stage III	4 (8%)	2.33 (1.29–4.21)		3.20 (0.67–15.14)	0.143
Stage IV	19 (39%)	2.17 (1.60–2.94)		58.37 (8.66–393.62)	<0.001*
Tumor size					
<4 cm	10 (20%)	Ref.	0.9		
≥4 cm	41 (80%)	1.05 (0.43–2.57)			
CEA serum levels					
<5 ng	21 (48%)	Ref.	0.008*	Ref.	0.188
≥5 ng	23 (52%)	2.81 (1.28–6.16)		0.38 (0.09–1.61)	
Microsatellite instability					
No	42 (84%)	Ref.	0.8		
Yes	8 (16%)	1.12 (0.48–2.62)			
EGFR					
<35%	36 (73%)	Ref.	0.005*	Ref.	0.044*
≥35%	13 (27%)	3.15 (1.36–7.33)		0.31 (0.10–0.97)	
β-catenin					
Negative	29 (58%)	Ref.	0.6		
Positive	21 (42%)	0.82 (0.39–1.72)			
CEA					
Negative	11 (22%)	Ref.	1		
Positive	39 (78%)	0.99 (0.45–2.16)			
c-Myc					
<10%	9 (19%)	Ref.	0.6		
≥10%	39 (81%)	0.80 (0.32–1.96)			
Her2					
Negative	49 (98%)	NA	NA		
3+	1 (2%)				
Ki-67					
<50%	34 (69%)	Ref.	1		
≥50%	15 (31%)	1.00 (0.45–2.20)			
p53					
0	13 (26%)	Ref.	0.7		
<10%	15 (30%)	1.36 (0.53–3.48)			
≥10%	22 (44%)	0.99 (0.61–1.59)			

Results expressed as number of cases (percentage). HR: hazard ratio. Ref.: reference group for HR calculation. *Statistically significant at p < 0.05. NA: not available, too few data elements in one group.

Stats for multivariant model: Concordance = 0.803 (se = 0.057); R^2^ = 0.495; Likelihood ratio test = 28.67 (p < 0.001); Wald test = 26.02 (p < 0.001); Score test = 37.55 (p < 0.001).

Multivariate analysis of the prognostic factors for OS revealed two variables that independently predicted an adverse outcome: EGFR expression (*p* = 0.047) and pTNM stage (*p* < 0.001) (Table 3).

### EGFR protein expression and copy number

**Figure 2:**
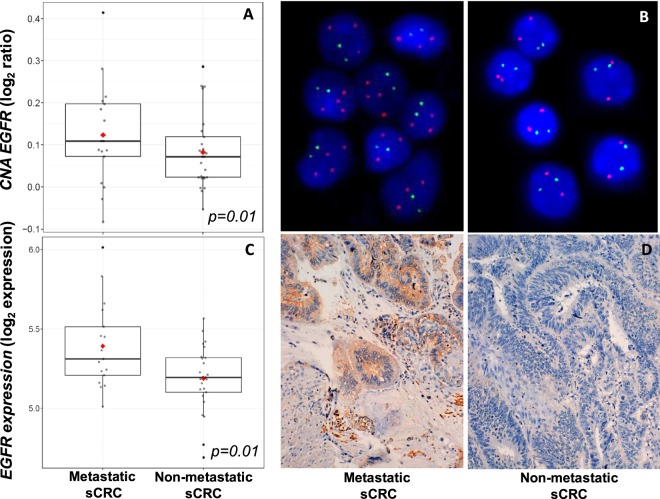
Copy number alterations (CNAs) and EGFR gene expression levels detected in primary tumors from patients with metastatic and non-metastatic sCRC. CNA status assessed by Affymetrix 500‐K single nucleotide polymorphism (SNP)‐array platform (panel A) and FISH techniques [probes for identifying chromosome 7 centromere (7p11; green spots) and EGFR gene (7p11.2; red spots)] (panel B). EGFR gene expression profile analyzed by oligonucleotide arrays (Affymetrix PrimeView Human Gene Expression microarray) (panel C) and immunostaining techniques (20x) (panel D). Panel (B,D) correspond to a sample of the same patient.

**Figure 3:**
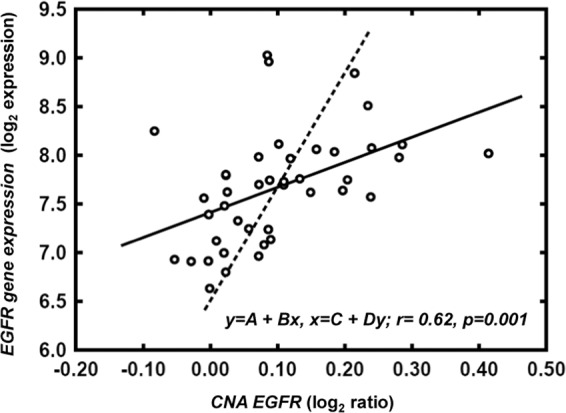
Correlation between EGFR gene expression using oligonucleotide arrays and CNA by SNP arrays in patients with sporadic colorectal cancer at diagnosis. The graphs show the regression lines for Y as a function of X (solid line) and for X as a function of Y (dashed line). If these regression lines are approximately perpendicular, it indicates that X and Y are not linearly correlated. The closer the lines, the greater the correlation.

There was a significant association between EGFR IHC positivity, EGFR gene copy number, as determined by FISH, and SNP arrays and GEP, detected by oligonucleotide arrays, between metastatic and non-metastatic CRC cases (Fig. 2) and the whole series (Table 4 and Fig. 3). Most tumors with a low level of EGFR IHC expression (<35%) were disomic (75% of cases, as revealed by FISH), had a normal profile (68% of cases, demonstrated by SNP arrays) and a low level of expression of EGFR mRNA determined by oligonucleotide arrays (<4 log_2_ expression; 55% of cases), whereas all tumors with EGFR IHC overexpression ≥35%) showed amplifications (57%) or a high proportion of polysomies (43%) with FISH, and most exhibited EGFR gains (71%) in the SNP analysis. In addition, all tumors with a high degree of EGFR IHC positivity (≥35%) were overexpressed in oligonucleotide arrays (>4 log_2_ expression).

**Table 4:** Correlation between EGFR immunohistochemical (IHC) expression, EGFR gene copy number, determined by FISH techniques and SNP-arrays, and GEP, detected by oligonucleotide arrays, in patients with sporadic colorectal cancer at diagnosis.

	EGFR IHC expression		*p*
	<35%	≥35%	
**FISH**			
Normal	27 (75%)	0	<0.001
Amplification	0	8 (57%)	
Polysomy	9 (25%)	6 (43%)	
**SNPs**			
Normal	21 (68%)	2 (29%)	0.07
Gain	10 (32%)	5 (71%)	
GEP*			
<4	17 (55%)	0	0.009
≥4	14 (45%)	7 (100%)	

FISH: interphase fluorescence *in situ* hybridization; SNP: single nucleotide polymorphism;

GEP: gene expression profile; IHC: immunohistochemistry; Results expressed as number

of cases and percentage in parentheses.

*Log_2_ expression.

SNPs arrays and GEP studies were performed in 38 cases due to sample restrictions

The coefficient of concordance (Wt) between EGFR protein expression and copy number by arrays studies was of 0.79 (p = 0.01) for EGFR log2 gene expression vs. *EGFR* copy number (logRatio), which indicates a good agreement between both measurements

Given the immunohistochemical criteria for the expression of p53 previously established by Kaserer *et al*.^12^, we note that most patients with loss or mutation at the p53 level (18/35 cases) showed polysomies or *EGRF* amplification (Supplementary Table 1).

### miRNA genes potentially regulate EGFR gene expression

In order to determine the impact of the miRNAs on EGFR gene expression in sCRC tumors, 134 candidate miRNAs targeting EGFR expression predicted from the miRDB database were combined to investigate possible correlations between miRNAs and the EGFR gene transcript in metastatic and non-metastatic tumors. Evaluation of each potential miRNA-mRNA pair targeting EGFR genes identified possible interactions for two negatively correlated (absolute R^2^ ≥ 0.71; *p* < 0.0001) pairs of miRNA-mRNA genes. Using available miRNA target prediction algorithms and databases indicated that, such interactions corresponded to only two predictable interactions for the inversely correlated miRNA-mRNA pair (Table 5), miR-134 and miR-4328, in non-metastatic and metastatic tumors, respectively.

**Table 5:** miRNA-mRNA interactions identified in non-metastatic and metastatic colorectal patients by Pearson correlation analysis of the expression signal identified for EGFR transcripts, detected by the Affymetrix PrimeView human gene expression array and the microRNA 3.0 expression array.

miRNA	R^2^	*p*	Classification of interaction	Source of validation/prediction
**Non-metastatic tumors**				
hsa-miR-134_st	−0.72	<0.001	Predicted	miRWalk, miRDB, MiRanda, DIANAmT
hsa-miR-3144-3p	−0.53	0.006	Predicted	miRWalk, miRDB, TargetScan
hsa-miR-4659a-5p	−0.52	0.007	Predicted	miRWalk, miRDB
hsa-miR-133b	−0.51	0.009	Predicted	miRDB
hsa-miR-651_st	−0.47	0.01	Predicted	miRDB, TargetScan
hsa-miR-27a-star_st	−0.46	0.02	Predicted	miRDB, MiRanda, TargetScan
hsa-miR-4476	−0.42	0.03	Predicted	miRDB, miRWalk,TargetScan
hsa-miR-155_st	−0.42	0.03	Predicted	miRDB, TargetScan
hsa-miR-1227_st	−0.42	0.03	Predicted	miRDB, miRWalk, TargetScan
hsa-miR-3126-3p	−0.42	0.03	Predicted	miRDB, TargetScan
**Metastatic tumors**				
hsa-miR-4328	−0.71	0.001	Predicted	miRDB, TargetScan, MiRanda, DIANAmT
hsa-miR-651_st	−0.64	0.006	Predicted	miRDB, TargetScan, miRWalk
hsa-miR-3616-5p	−0.63	0.006	Predicted	miRDB, TargetScan
hsa-miR-876-3p	−0.62	0.008	Predicted	miRDB, miRWalk
hsa-miR-1233_st	−0.61	0.01	Predicted	miRDB, TargetScan, miRWalk
hsa-miR-548e_st	−0.60	0.01	Predicted	miRDB
hsa-mir-302c_st	−0.59	0.01	Predicted	miRDB
hsa-mir-373_st	−0.55	0.02	Predicted	miRDB, TargetScan, miRWalk
hsa-miR-3686	−0.55	0.02	Predicted	miRDB, TargetScan, miRWalk
hsa-miR-19a-star_st	−0.55	0.02	Predicted	miRDB, TargetScan, miRWalk
hsa-miR-646	−0.54	0.03	Predicted	miRDB, TargetScan, miRWalk
hsa-miR-4504	−0.53	0.03	Predicted	miRDB, TargetScan
hsa-mir-141_st	−0.52	0.03	Predicted	miRDB, TargetScan, miRWalk
hsa-miR-4659a-5p	−0.52	0.03	Predicted	miRDB, TargetScan

R^2^: Pearson correlation coefficient.

**Figure 4:**
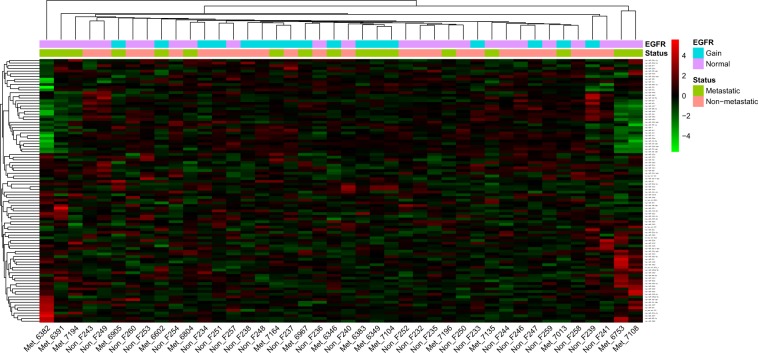
Hierarchical cluster analysis based on EGFR expression regulated by 98 miRNAs present in our array (out of 134 miRNAs total candidates) of the non-metastatic (n = 25) and metastatic cases (n = 26) included in the study.

Area under the receiver operating characteristic curve (AUROC) from the generalized linear model (GLM) reached a value of 0.70 considering a model with the miR-134 and miR-4328, that is, our GLM model has a chance of 70% of distinguishing between metastatic and non-metastatic patients based on the expression of those two microRNAs. Other machine learning methods used for this purpose, such as SVM (0.58), KNN (0.58) and RF (0.46), showed lower values of AUCROC indicating a weaker discriminating power. Conversely, the hierarchical cluster analysis based on EGFR expression regulated by 98 miRNAs present in our array (out of 134 miRNAs total candidates) was not able to discriminate among metastatic and non-metastatic tumors, as shown in Fig. 4.

### Validation of the clinical impact of the EGFR gene expression levels and its correlation with CNA in an independent series of patients

**Figure 5:**
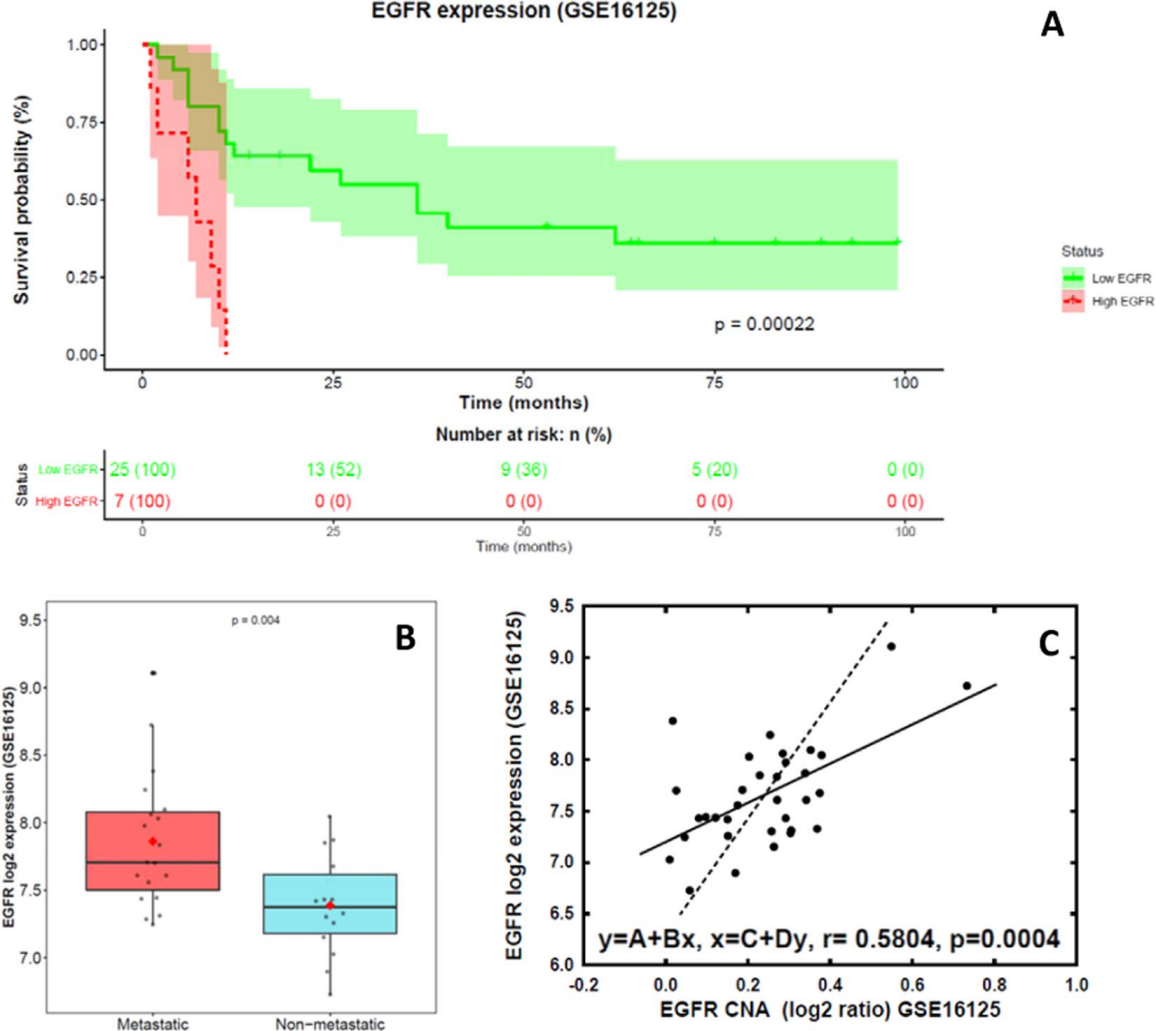
Validation of the impact of EGFR expression on overall survival in an independent series of sCRC patients from the GEO database (n  =  32): panel (A), EGFR expression showing the impact on overall survival; panel (B), EGFR gene expression levels detected in primary tumors from patients with metastatic and non-metastatic sCRC; and panel (C), correlation between EGFR gene expression using oligonucleotide arrays and CNA by SNP arrays in patients with sporadic colorectal cancer at diagnosis.

In order to confirm the clinical significance of the EGFR gene expression, we investigated its prognostic impact in an independent series of colorectal cancer patients from the public GEO database (n = 32). Noteworthy, also in this new series, patients whose tumors with high level of expression of EGFR mRNA determined by oligonucleotide arrays (>8 log_2_ expression; 22% of cases), were found to have an inferior clinical outcome than those harboring low levels (≤8 log_2_ expression; 78% of cases). In line with our observations, these results also confirmed the existence of significant differences in the expression of the EGFR gene between non-metastatic (n = 14) and metastatic cases (n = 19). In addition, we also found a good correlation between EGFR gene expression levels and CNA (Fig. 5). These results support the observations of our dataset and confirm the prognostic impact of EGFR expression.

## Discussion

Sporadic colorectal cancer (sCRC) patients who do not exhibit or develop distant metastasis can often be cured by surgical resection of the primary tumor, and the optional administration of adjuvant therapy. However, the chances of a cure are dramatically reduced when metastasis to the liver or other organs occurs. Even though we now have a much better understanding of the genetic mechanisms that control the early stages of familial^13^ and sCRC^14^, the role of IHC in stratifying patient risk is still controversial^15–23^. In this study, we investigated the immunohistochemical profile of primary tumors from metastatic and non-metastatic sCRC patients. To avoid false-negative non-metastatic cases, only sCRCs with a relatively long follow-up (median, 146 months) were included in the non-metastatic tumor group. Similarly, only liver metastatic patients whose primary and metastatic tumors had been completely resected were classified in the metastatic patient group.

Antibodies targeting the proteins most frequently involved in sCRC^24,25^ were specifically applied for the immunohistochemical characterization of the two patient groups. Previous observations showing that liver metastatic and non-metastatic sCRCs^16,26^ share a similar pattern of protein expression, as revealed by IHC, were confirmed by our finding of similar immunohistochemical profiles in metastatic and non-metastatic liver tumors for the expression of most of the antibodies studied (e.g., high levels of c-Myc and p53, Her2 negativity, and Ki-67 expression ≤50% in most cases). Conversely, MLH1, PMS2, CEA and EGFR expression was markedly more common in, and in same cases exclusive to, liver metastatic tumors. Our findings imply that these four proteins may play a role in the metastasis of sCRC to the liver.

Earlier reports of immunohistochemical analyses of metastatic disease in colorectal tumors demostrated the presence of microsatellite instability (MSI) arising from the loss of DNA mismatch repair (MMR) expression in 15% of sCRC tumors^27,28^. Consistent with the findings of other studies, and using similar methods, we found MSI to be present in 16% of the sCRC cases studied. The MSI detection rate was also significantly higher in our non-metastatic patients than in liver metastatic cases a pattern that has been described by other groups^28–30^. Several studies have shown that the microsatellite profile in sCRC provides useful prognostic information^29^, indicating that patients with MSI neoplasms have better OS and a modified response to conventional chemotherapy^31^. However, the better prognosis of MSI carcinomas is not established beyond doubt; other studies have shown that these tumors behave similary, from a clinical point of view, to microsatellite-stable (MSS) carcinomas, and no clinical benefit has been observed except in stage II tumors^32^. There is still no definitive explanation of the prognostic advantage accruing from MSI, although intense lymphocytic infiltration, an increased rate of apoptosis, and infrequent allelic loss or mutation of *TP53*, *DCC*, *KRAS* and *BRAF* in MSI colorectal cancer are thought by some to determinate their clinical behavior^33–35^.

The consensus statement of the College of American Pathologists published in 1999 indicated that pathological TNM stage, extramural venous invasion, and preoperative CEA serum level are the most important category I prognostic factors^36^. It is well established that the preoperative CEA serum level is an important prognostic factor^36,37^, whereby levels greater than 5 ng/ml are related to worse prognosis. In fact, our recent study reported highly prevalent, abnormally high CEA serum levels (≥7.5 ng/ml) in the great majority of primary sCRC patients who had synchronous liver metastasis^6^. Consistent with the findings of previous studies using IHC techniques^23^, we observed a significantly higher level of CEA expression in patients with metastatic tumors than in those with non-metastatic tumors. CEA is involved in cell adhesion, protecting cells from anoikis (apoptosis induced by the loss of anchoring of the cell to the extracellular matrix)^38^, which favors the cell’s metastatic potential. In addition, CEA can bind to Kupffer cells^39^, modulate the inflammatory response in the liver, and protect tumor cells from oxygen radicals^40^. *In vitro* studies have shown resistance of cells expressing CEA to lysis induced by activated killer cells (LAK cells)^41^. These biological functions of CEA may explain why tumors with stronger expression have greater metastatic potential.

In addition to CEA expression associated with the metastatic process, we also found EGFR expression to be an independent prognostic factor of disease outcome, as previously observed^42,43^. Previous studies employing IHC analysis^42,43^ have shown an association between EGFR expression and liver metastasis in sCRC patients; here, we also found that EGFR-positive tumors had lymph node metastases and a higher TNM category at diagnosis. Similarly, Goos *et al*.^44^ reported that, in a series of 323 patients with metastatic CRC, those who strongly expressed EGFR had a worse prognosis. Other studies have found a correlation between EGFR expression and more advanced disease^43,45,46^, metastatic spread^23,47,48^ and worse prognosis^49^. In addition, Du P *et al*.^48^ have suggested that EGFR is important in the angiogenesis in early tumors. EGFR is linked to colorectal carcinoma progression, and so is a widely used prognostic factor. However, other authors have reported controversial results from their IHC assessment of EGFR expression and its correlation with sCRC prognosis. Tsai *et al*.^50^ found no differences in EGFR expression between tumors of various grades and stages, or in survival, despite analyzing a large series of patients (n = 150). This could be because EGFR-positive cases were classified into three categories (1+, 2+ and 3+), rather than being measured as the percentage of positive tumor cells, as we have done in the present study.

Genomic heterogeneity is a weidely acknowledged challenge in the treatment of colorectal tumors. To compare the results obtained by techniques that analyze individual cells with those from methods based on the entire genome, we extracted the DNA/RNA from the same tumor tissue analyzed in the TMAs through microdissections of the paraffin-embedded tissue. In this way, we found significant correlations between EGFR staining results, and those of oligonucleotide expression arrays, FISH analysis and SNP arrays. This is the first study in which the immunohistochemical expression of EGFR has been validated by three molecular techniques in metastatic and non-metastatic tumors. In accordance with other studies, EGFR overexpression was frequently accompanied by gene amplification and/or a high frequency of chromosome 7 polysomies, especially in metastatic colorectal tumors, as found by other researchers^47,51–53^. Two reviews of the data have shown that patients with sCRC that has been diagnosed as EGFR-positive by FISH also responded to cetuximab monotherapy treatment^52,53^. In fact, the dose of cetuximab that was sufficient to inhibit proliferation in mCRC cell populations with amplified numbers of *EGFR* copies had no effect on cell populations without EGFR amplification. Moroni M *et al*. found that eight of nine patients with objective responses who could be assessed by FISH had a higher EGFR copy number, suggesting that selection of candidates for anti-EGFR therapy may be based on *EGFR* gene copy number^52^. Our findings show that transcriptional upregulation, abnormal receptor structure secondary to genetic alterations (e.g., mutation and polymorphism) gene amplification or specific miRNA levels could be responsible for EGFR overexpression, and therefore predictors of disease prognosis^54,55^.

Here, we also identified miR-134 and miR-4328 as negatively regulated EGFR targets in non-metastatic and metastatic tumors, respectively. Previous studies have confirmed that both miRNAs have important roles in the progression of sCRC. These studies of the EGFR-miRNA regulation network draw attention to the possibilities of using miRNA-based therapy to target EGFR, in addition to employed tyrosine kinase inhibitors and classical monoclonal antibodies for EGFR-targeted therapies,. miRNA-134 has important roles in cancer, such as regulating migration^56^, invasion^56^, cell proliferation^56,57^, and the epithelial–mesenchymal transition^58,59^. Qin *et al*.^60^, using intratumoral injection of miR-134, observed a reduction in the expression of EGFR in non-small-cell lung carcinoma (NSCLC) cell lines, suggesting that the use of miR-134 could be a potential strategy for EGFR-targeted therapy. Sherien *et al*.^61^ showed that miRNA-134 is a potential tumor suppressor miRNA and could be fundamental to the suppression of colorectal cancer tumorigenesis since it is able to regulate the EGFR signaling cascade in a coordinated fashion by independently targeting EGFR and PIK3CA. In metastatic tumors, EGFR overexpression was more frequently detectedas a consequence of gene amplification or a high frequency of polysomies. Interestingly, interactions of the inversely correlated miRNA-mRNA pairs in the metastatic patient series revealed miRNA-4328 to be a possible regulator of *EGFR* expression. Information is available about miRNA-4328 in CRC patients; it has been previously described as an EGFR regulator in EGFR-mutated lung adenocarcinomas^62^. However, further GEP and functional studies, and direct comparison of non-metastatic and metastatic tumors are needed to confirm our observations and to provide deeper insight into the role of miRNA-4328 in metastatic CRC patients.

In summary, in the present study, we show that EGFR protein expression and copy number are closely related. EGFR expression is an independent adverse prognostic factor of OS in sCRC patients. The expression of the *EGFR* gene could be regulated by amplification mechanisms or a high frequency of polysomies, as is often observed in metastatic tumors, or by miRNAs (i.e., miRNA-134) in non-metastatic tumors. We also showed that the possible involvement of EGFR expression in sCRC patients with liver metastasis is inherently related to greater metastatic potential and worse outcome, which provides additional prognostic information about the pTNM stage. Additional prospective studies, in larger series, are needed to confirm the utility of the proposed predictive model.

## Materials and Methods

### Approval

The study was approved by the local ethics committee of the University Hospital of Salamanca (Salamanca, España).

### Accodance

All procedures performed in studies involving human participants were in accordance with the ethical standards of the institutional and/or national research committee and with the 1964 Helsinki declaration and its later amendments or comparable ethical standards.

### Informed consent

Informed consent was obtained from all participants before entering the study,

### Patients and samples

Tissue specimens from 51 sCRC patients who had undergone surgical resection of primary tumor tissues between June 2000 and September 2007 in the Department of Surgery of the University Hospital of Salamanca, Salamanca, Spain, were included in the study, prior to administering any cytotoxic therapy.

In all cases, tumor were diagnosed and classified according to the criteria of the AJCC^63^ [12]. The median follow-up at the moment of closing the study was 103 months for the whole series (range: 1–172 months). The median follow-up for metastatic patients was 49 months (range: 1–153 months) and 146 months (range: 54–172) for non-metastatic patients. About half the patients (n = 25; 49%) developed liver metastases during the first 8 months after colorectal surgery (n = 16), or later, during follow-up (n = 9). The other 26 patients (51%) were non-metastatic sCRC cases, selected on the basis of the absence of metastatic dissemination and after a minimum follow-up of 5 years. Patient clinical, laboratory and follow-up data are summarized in Table 6.

**Table 6:** Clinical and biological characteristics of patients with metastatic (n = 25) and non-metastatic (n = 26) sporadic colorectal cancer (sCRC) at diagnosis.

Variable	Non-metastatic sCRC (n = 26)	Metastatic sCRC (n = 25)	*p*	Total (n = 51)
Age (years)*	67 (38–83)	67 (48–79)	NS	67 (38–83)
**Gender**				
Female	7 (27%)	6 (24%)	NS	13 (25%)
Male	19 (73%)	19 (76%)		38 (75%)
**Site of primary tumor**				
Right colon	9 (35%)	4 (16%)	0.020	13 (26%)
Left colon	14 (54%)	10 (40%)		24 (47%)
Rectum	3 (11%)	11 (44%)		14 (27%)
**Histopathological grade**				
Well differentiated	19 (73%)	16 (64%)		35 (69%)
Moderately differentiated	6 (23%)	8 (32%)	NS	14 (27%)
Poorly differentiated	1 (4%)	1 (4%)		2 (4%)
**Histopathological tumor classification**				
pT1	1 (4%)	0 (0%)	0.060	2 (4%)
pT2	6 (23%)	1 (4%)		6 (11%)
pT3	11 (42%)	19 (76%)		30 (59%)
pT4	8 (31%)	5 (20%)		13 (26%)
**Lymph node involvement**				
pN0	26 (100%)	9 (36%)	<0.001	35 (69%)
pN1	0 (0%)	11 (44%)		11 (21%)
pN2	0 (0%)	5 (20%)		5 (10%)
**TNM stage at diagnosis**				
I	6 (23%)	1 (4%)	<0.001	7 (14%)
IIA	1 (4%)	2 (8%)		3 (6%)
IIB	17 (65%)	0 (0%)		17 (33%)
IIIA	0 (0%)	0 (0%)		0 (0%)
IIIB	2 (8%)	1 (4%)		3 (6%)
IIIC	0 (0%)	1 (4%)		1 (2%)
IV	0 (0%)	20 (80%)		20 (39%)
CEA serum levels*	4.18 (0.60–17.96)	269.22 (0.84–1484)	<0.001	118.63 (0.60–1484)
Number of deaths**	2 (8%)	13 (72%)	<0.001	15 (34%)
OS (months)**	99.69 (71–124)	31.61 (7–96)	<0.001	71.84 (7–124)

Results expressed as number of cases (percentage), except * as median (range). **Survival rate calculated for 44 patients (26 non-metastatic and 18 metastatic). CEA: carcinoembryogenic antigen; OS: overall survival; NS, no statistically significant differences found between groups (*p* > 0.05).

### Patient characteristics

Overall, 51 patients diagnosed with sCRC at the University Hospital of Salamanca (38 males and 13 females; median age, 67 years, range: 38 to 83 years) were studied. Histologically, all cases were adenocarcinomas. By tumor grade, 35, 14 and 2 cases were classified as well, moderately and poorly differentiated carcinomas, respectively. In all cases, histopathological grade was systematically confirmed by a second, independent evaluation by another experienced pathologist. The most relevant clinical and laboratory data for each individual sCRC patient studied are summarized in Table 6.

All patients underwent complete surgical tumor resection (R0). sCRC cases with liver metastases were most frequently located in the rectum (*p* = 0.02), and tended to show a higher frequency of lymph node metastases (*p* < 0.001) and abnormally higher CEA serum levels (*p* < 0.001) than non-metastatic patients (Table 1). From the prognostic point of view, sCRC with liver metastases also had a higher frequency of deaths associated with significantly shorter patient OS (median, 30 *vs*. 100 months, respectively; *p* < 0.001). By contrast, no significant differences were found between liver metastatic and non-metastatic CRC cases with respect to patient age, gender and histological grade (Table 6).

### Tissue microarray (TMA)

**Figure 6:**
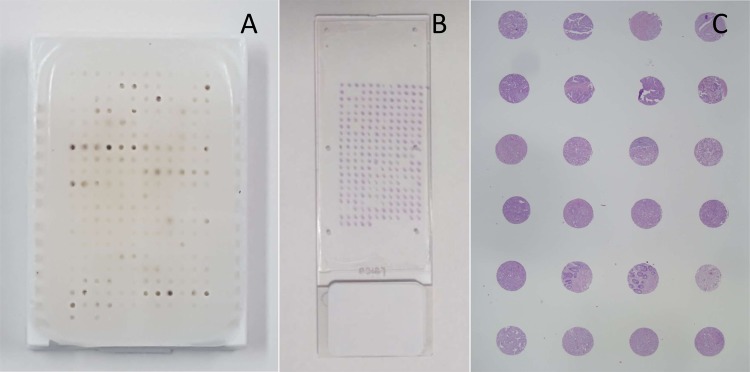
TMA: (**A**) TMA paraffin block; (**B**) TMA slide, hematoxylin-eosin; (**C**) hematoxylin-eosin panoramic view, 3-µm section.

Paraffin blocks containing formalin-fixed primary tumors were produced for all patients. For each of these, a 0.6-mm diameter core biopsy was taken from the tumor in the paraffin blocks with tissue microarray apparatus. Tissue cores of each specimen were arranged on recipient paraffin blocks and embedded. Two colon cancer TMAs were prepared using a Beecher MTA-1 Manual Tissue Microarrayer, incorporating tissue selected by a pathologist from histological sections. One TMA contained samples from metastatic tumors and the other contained non-metastatic cases. Cores of normal tissue were added and all tumor samples were included in triplicate in both TMAs (Fig. 6).

### Immunohistochemistry

4-μm-thick sections of the TMA blocks were immunohistochemically stained. Paraffin sections were deparaffinized in xylene and rehydrated in an ethanol series. Microwave-induced antigen retrieval was carried out in 0.01 M citrate buffer, pH 6.0. Endogenous peroxidase activity was curtailed using 0.3% hydrogen peroxide in methanol for 15 min. Antibodies and reagents were included automatically using the Leica BOND-III processor (A. Menarini Diagnostics, San Diego, CA), following previously published standard protocols^64^. Sections were counterstained with hematoxylin, dehydrated and mounted. Primary antibodies, dilutions, manufacturers,interpretations and positive controls used are specified in Supplementary Table 2. Based on a literature review, we established different cutoffs for each antibody used (c-Myc: <10%, ≥10%^22^; Her2: 0/1+, 2+, 3+^21^; p53: 0, <10%, ≥10%^12^; ß-catenin: <30%, ≥30%^25^; CEA: no stained cells or non-specific stain, any specific cytoplasmic stain^20,41^; EGFR: 0, 1–10%, 10–35%,>35%^44^; Ki67: <50%, ≥50%^17,18^.

Cases in which there was no tumor sample that could be assessed by immunohistochemical techniques (due to detachment of the tissue in fron the slide, tumor depletion, or a poor outcome from the technique) were excluded and not considered in the statistical analysis (Table 1).

We found no significant variability between the three tumor samples of the patient included in the TMAs.

### FISH analysis

Separate FISH analyses were performed for each TMA, one containing samples from metastatic tumors and the other from non-metastatic cases. The TMA slides were preheated overnight at 60 °C, then deparaffinized in xylene and ethanol. The slides were submerged in Vysis Paraffin Pretreatment Reagent (Vysis; Downers Grove, IL) for 13 min at 80 °C in a waterbath. Sections were rinsed with deionized water before treating the slides with protease solution (250 mg pepsin + 62.5 ml 0.2 N HCl, pH 1.0) for 13 min at 37 °C in a waterbath. After rinsing and air-drying, 20 µl of probe was added to each TMA slide. A set of two probes (LSI-EGFR, spectrum orange; CEP-7, spectrum green) (Vysis Inc., Downers Grove, IL) was used. A coverslip was placed on each slide and sealed in place with rubber cement. Slides were then denatured for 5 min at 75 °C, and hybridized overnight at 37 °C in a Hybrite thermocycler (Vysis). Slides underwent post-hybridization washing consisting of in 2 X SSC with 0.3% NP-40 at 73 °C in a waterbath for 1 min. The slides were air-dried in darkness and counterstained with 20 µl of DAPI (Sigma, St. Louis, MO). 5 µl of Vectashield (Vector Laboratories, Burlingame, CA) was used as an antifading agent. The number of hybridization spots per nuclei of ≥ 200 cells per sample was quantified using a BX60 fluorescence microscope (Olympus, Hamburg, Germany) equipped with a 100× oil objective. *EGFR* gene amplification was defined as an *EGFR*/CEP7 ratio ≥ 2, in accordance with the manufacturer’s recommendations. In all cases it was possible to quantify at least 200 tumor nuclei. Two pathologists independently interpreted the results, and concurred completely in their observations.

Once the TMAs had been prepared, consecutive sections of paraffin-embedded tissue samples were microdissected by an experienced pathologist. DNA/RNA was extracted and isolated using a Maxwell® 16 System for Genomic DNA/RNA Extraction (Promega, Mannheim, Germany) and quantified using a Qubit dsDNA BR and RNA assay (Invitrogen, Life Technologies, CA, USA)

### Identification of copy number alterations by SNP arrays

Data from two 250-K Affymetrix SNP mapping arrays (NspI and StyI SNP arrays; Affymetrix, Santa Clara, CA) previously reported by us^8^ were used to analyze EGFR CNAs. The aroma.affymetrix algorithm was used, following the CRMA version 2 method in R^65^ (R Foundation for Statistical Computing, Vienna, Austria; available at: http://www.aroma-project.org, accessed 18 March 2014). The steps taken to normalize the arrays were those described by Munoz-Bellvis *et al*.^66^. Data from the 250-K StyI and 250-K NspI arrays were then compiled in a single database. Raw copy numbers were recalculated for each group as transformed log_2_ values of the following ratios: group 1 primary tumor = normal PB; group 2 primary tumor=normal PB; and group 1 primary tumor = group 2 primary tumor.

The criteria used to define single-point copy number changes in the EGFR gene were based on values of *p* < 0.01 for at least seven markers for each DNA segment, and smoothed values were used to assign median segment values to each probe. To identify CNAs (gains or losses), a threshold was declared based on the changes in the fluorescence intensity of sequential DNA segments for primary tumor *versus* PB (log_2_ ratio cutoff values of>0.09 and <20.09 for gains and losses, respectively). Moderated t tests were used to identify significant differences in the mean copy number of metastatic and non-metastatic samples (FDR-corrected *p* < 0.05).

### Gene expression profile microarray studies

Data from two expression arrays, Affymetrix PrimeView Human Gene Expression and the microRNA 3.0 Expression arrays (Santa Clara, CA), previously reported by us^67^ were used to analyze EGFR expression. Raw GEP data were obtained from the Gene Expression Omnibus (GEO) database (accession number GSE81582). The data are based on sets of 49,395 and 5,683 probes, respectively, for the Affymetrix PrimeView Human Gene Expression microarray and the microRNA 3.0 microarray. For data analysis, raw GEP data were normalized by applying the Robust Multi-array Average (RMA) algorithm, which includes sequential background correction, intra- and inter-microarray well normalization, probe set summarization and calculation of expression signals^68^. Samples and the EGFR gene were classified in an unsupervised manner by multi-dimensional scaling (MDS) and hierarchical clustering analysis (HCA) based on the expression signal of each gene of each probe set, using Simfit software (http://www.simfit.org.uk/). Clustering was carried out assuming Euclidean distances and employing the linkage method group average. Genes differentially expressed (miRNA and mRNA) in tumoral and non-tumoral samples were identified by estimating the supervised two-class unpaired significance of microarray (SAM)^69^ based on a cutoff value of q ≤ 0.01 and an absolute ≥2.0-fold change cutoff.

In addition, we have performed a concordance analysis between EGFR protein expression and copy number by arrays studies using the Kendall’s (W) concordance coefficient through the DescTools R package (v. 0.99.31).

miRNA candidates acting as regulators of the EGFR gene in colorectal samples were identified by Pearson correlation analysis, which determined the significant associations between EGFR gene transcripts and deregulated miRNA in primary colorectal tumors, compared with non-tumoral colorectal tissue. The psych R-package, based on an adjusted FDR of ≤0.05 was used. We analyzed 134 candidate miRNAs targeting EGFR expression predicted from the miRDB database. Each potential miRNA-mRNA interaction identified was evaluated against the TarBase 8.0 and miRWalk databases of experimentally validated miRNA interactions, and the DIANA-microT-CDS v5.0, miRWalk-database, TargetScanHuman and miRecords miRNA target prediction tools^70,71^.

Hierarchical dendrograms were performed in R using the gplots (v.3.0.1.2) and the pheatmap (v. 1.0.12) packages. Dendrograms were performed using the Euclidean distance as distance measure and the group average as linkage method (Fig. 4).

A ROC analysis was included to further support the power of miR-134 and miR-4328 in discriminating the non-metastatic and metastatic tumors. Area under ROC curve (AUROC) analysis was performed using the Generalized Linear Models (GLM), Support Vector Machines (SVM), K-Nearest Neighbors (KNN) and Random Forest (RF) classification methods. Analyses were performed in R using the randomForest (v. 4.6–14), e1071 (v. 1.7–3) and caret (v. 6.0–85) packages by k-fold cross validation considering 5 folds and 1000 repeats. The optimal kernel for SVM analysis was determined using the OptimClassifier (v.0.1.5) package.

### Mutation analyses using low-density microarrays

Following their histopathological diagnosis, each tumor was tested for the presence of mutations of the *KRAS*, *NRAS* and *BRAF* genes. A multiplex allele-specific PCR-based assay was used for this purpose that assesses 44 mutations in *KRAS* codons 12, 13, 59, 61, 117 and 146 (G12A, G12C, G12D, G12R, G12S, G12V, G13D, A59E, A59G, A59T, Q61K (C > A), Q61K (C > AA), Q61L, Q61R, Q61H(A > T), Q61H(A > C), K117N(A > C), K117(A > T), A146P, A146V, and A146T), *NRAS* codons 12, 13, 59, 61, 117 and 146 (G12D, G12C, G12S, G12A, G12V, G13D, G13R, G13V, A59T, Q61K, Q61R, Q61L, Q61H(A > C), Q61H(A > T), K117N(G > C), K117N(G > T), A146T and A146V) and *BRAF* codon 600 (V600E(T > A), V600E(G > AA), V600D, V600K and V600R). 51 assays were carried out using the CLART^®^ CMA.KRAS.BRAF and CLART^®^ CMA.NRAS.iKRAS kits (Genómica SAU Technology, Madrid, Spain) following the manufacturer’s instructions. These are based on PCR amplification and array hybridization, respectively, with a variety of probes.

### External validation of the prognostic impact of EGFR gene expression levels and its correlation with CNA

External validation of EGFR gene expression levels found to be differentially expressed in our series between non-metastatic and metastatic tumors, was performed in a group of previously reported sCRC patients (n = 32) from whom GEP and CNA files and clinical data (Affymetrix, CA) are publicly available at the GEO database with accession number GSE16125 (Fig. 5)^72^.

### Statistical analyses

The mean, standard deviation (SD) and range of all continuous variables were calculated in SPSS v.22 (IBM Corp., Armonk, NY, USA); dichotomous variables were reported as frequencies and percentages. To evaluate the statistical significance of group differences, unpaired Student’s t and Mann-Whitney U tests were used for normally and non-normally distributed continuous variables, respectively. For dichotomous variables, the X2 test was used. Overall survival (OS) curves were plotted according to the Kaplan–Meier method. Statistical significance of the differences between survival curves was determined with one-sided log-rank tests. Prognostic factors for OS were identified by multivariate stepwise Cox regression, using forward selection, considering only those variables that had shown a significant association with OS in univariate analyses. Statistical significance was concluded for values of *p* (or, where appropriate, Pearson-corrected *p*) <0.05.

## Supplementary information

Supplementary information
