# Supplementary material for: Prognostic implications of EGFR protein expression in sporadic colorectal tumors: Correlation with copy number status, mRNA levels and miRNA regulation
Source: Sci Rep. 2020 Mar 13;10:4662. doi: 10.1038/s41598-020-61688-7 (PMC7070091; doi:10.1038/s41598-020-61688-7)
Supplement: Supplementary file 1 — Supplementary information [file 41598_2020_61688_MOESM1_ESM.pdf]

Title: Prognostic implications of EGFR protein expression in sporadic colorectal tumors: Correlation with copy number status, mRNA levels and miRNA regulation

Running title: Biomarkers in Colorectal Cancer

Authors: Sofía del Carmen MD[#1], Luís Antonio Corchete[2], Ruth Gervas[1], Alba Rodriguez[1], María Garcia MD[3], José Antonio Álcazar MD, PhD[4], Jacinto García MD, PhD[4], Oscar Bengoechea MD, PhD[1], Luis Muñoz Bellvis MD, PhD*[4], José María Sayagués PhD*[#1] and Mar Abad MD, PhD*[#1]

*These authors contributed equally to this work and should be considered senior last authors.
[#]These authors should be considered corresponding authors.

Affiliations: [1]Department of Pathology and IBSAL, University Hospital of Salamanca, Salamanca, Spain; [2]Cancer Research Center and Hematology Service (University Hospital of Salamanca), Salamanca, Spain; [3]Department of Oncology, University Hospital of Salamanca; [4]General and Gastrointestinal Surgery Service, University Hospital of Salamanca.

Declaration of conflict of interest: The authors declare no potential conflicts of interest with respect to the research, authorship and publication of this article.

Keywords: Colorectal cancer, EGFR, TNM, FISH, SNPs, GEP, immunohistochemistry.

Reprint requests to:

Maria del Mar Abad, MD, PhD
University Hospital of Salamanca
Department of Pathology and IBSAL
Paseo de San Vicente, 58-182
37007 Salamanca, Spain
Tel: +34 923 090470
E-mail: marabad@usal.es

José María Sayagués, PhD
Institute for Biomedical Research of Salamanca (IBSAL)
Paseo de San Vicente, 58-182
37007 Salamanca, Spain
Tel: +34 923 090470
E-mail: ppmari@usal.es

Supplementary Table 1. Gene transcripts differentially expressed in primary metastatic vs non-metastatic sCRC tumors (n=23 vs n=25, respectively) vs non- tumoral colorectal tissues (n=9).

Gene Symbol	Affymetrix ID code	MTX vs non-MTX		MTX vs non-tumoral		non-MTX vs non-tumoral	
		Fold Change	FDR	Fold Change	FDR	Fold Change	FDR
ACA10	ACA10_s_st	-2.15	0.034	3.83	<0.001	3.47	<0.001
ACA21	ACA21_st	-2.44	0.046	2.09	0.044	3.48	0.001
ACA24	ACA24_s_st	-4.22	0.007			-2.18	<0.001
ACA26	ACA26_st	-2.01	0.030	3.06	<0.001	3.48	<0.001
ACA52	ACA52_st	-2.12	0.023	3.01	<0.001	3.48	<0.001
ACA57	ACA57_st	-2.32	0.040			-2.36	0.002
ACTA2	ENSG00000107796_at	2.30	0.034	6.58	0.001	6.80	<0.001
ADGRG7	ENSG00000144820_at	-2.31	0.026			2.14	0.036
ADH1B	ENSG00000196616_at	2.39	0.039			2.20	0.021
AEBP1	ENSG00000106624_at	2.41	0.024			2.15	<0.001
AKAP12	ENSG00000131016_at	2.51	0.032	9.68	<0.001	6.09	<0.001
ANP32E	ENSG00000143401_at	-2.38	0.002	3.49	0.001	7.65	<0.001
ASPN	ENSG00000106819_at	2.15	0.038	-3.30	<0.001	-2.95	<0.001
BST2	ENSG00000130303_at	-2.66	0.048				
CASP1	ENSG00000137752_at	-2.24	0.025			2.26	0.018
CNN1	ENSG00000130176_at	2.23	0.038	7.07	<0.001	5.85	<0.001
COMP	ENSG00000105664_at	2.96	0.034	2.16	0.046		
CXCL3	ENSG00000163734_at	-2.60	0.015			2.35	0.004
CXCL5	ENSG00000163735_at	-4.14	0.019	9.91	<0.001	9.82	<0.001
CYP1B1	ENSG00000138061_at	2.23	0.040	2.24	0.040	2.15	0.029
DES	ENSG00000175084_at	2.63	0.046	6.48	<0.001	6.03	<0.001
FABP4	ENSG00000170323_at	6.06	0.029	3.11	0.040	5.80	0.001
FBXO32	ENSG00000156804_at	2.10	0.008	13.06	<0.001	6.35	0.001
FER1L4	ENSG00000088340_at	2.06	0.050			2.92	0.012
GRP	ENSG00000134443_at	2.39	0.023	-2.58	0.001	-2.26	0.004
HBII-115	HBII-115_st	-2.32	0.028	2.89	0.001	3.49	<0.001
HBII-180A	HBII-180A_x_st	-2.49	0.005	3.49	<0.001	3.49	<0.001
HBII-180C	HBII-180C_x_st	-2.40	0.024	3.57	0.001	3.49	0.001
HBII-85-26	HBII-85-26_st	-5.02	<0.001	-2.35	0.001		
HBII-85-6	HBII-85-6_x_st	-3.30	0.001				
HMCN1	ENSG00000143341_at	2.03	0.039			2.79	<0.001
hp_hsa-mir-3648	hp_hsa-mir-3648_st	-2.11	0.020	2.73	<0.001	3.52	<0.001
hp_hsa-mir-3676	hp_hsa-mir-3676_st	-3.62	0.007				
hp_hsa-mir-4449	hp_hsa-mir-4449_st	-2.64	0.002	2.40	0.005	3.53	<0.001
hp_hsa-mir-4634	hp_hsa-mir-4634_st	-2.02	0.034	2.65	0.015	3.54	0.001
hp_hsa-mir-548ai	hp_hsa-mir-548ai_st	-4.55	<0.001			3.61	<0.001
hsa-let-7f	hsa-let-7f_st	2.20	0.013	2.30	0.006	3.56	<0.001
hsa-miR-1180	hsa-miR-1180_st	-2.01	0.041			2.17	<0.001

hsa-miR-1202	hsa-miR-1202_st	-2.20	0.023	4.70	<0.001	3.57	<0.001
hsa-miR-122	hsa-miR-122_st	2.39	0.001	-2.24	0.005	-2.18	0.005
hsa-miR-1225-5p	hsa-miR-1225-5p_st	-2.46	0.030	2.80	<0.001	3.58	<0.001
hsa-miR-1231	hsa-miR-1231_st	-2.49	0.012	2.19	<0.001	3.59	<0.001
hsa-miR-1285	hsa-miR-1285_st	-2.14	0.012	2.61	0.002	3.59	<0.001
hsa-miR-1307	hsa-miR-1307_st	-3.99	0.005	2.81	<0.001	3.62	<0.001
hsa-miR-130b	hsa-miR-130b_st	-3.43	0.006	2.09	0.010	3.64	<0.001
hsa-miR-135b-star	hsa-miR-135b-star_st	-2.06	0.010	4.26	<0.001	3.67	<0.001
hsa-miR-1469	hsa-miR-1469_st	-2.04	0.008	2.26	<0.001	3.69	<0.001
hsa-miR-146a	hsa-miR-146a_st	-2.51	0.018	2.32	0.001	2.66	<0.001
hsa-miR-150-star	hsa-miR-150-star_st	-2.65	0.011	15.90	<0.001	3.69	0.015
hsa-miR-17-star	hsa-miR-17-star_st	-2.65	0.018	2.48	0.011	3.74	<0.001
hsa-miR-18a-star	hsa-miR-18a-star_st	-2.24	0.021	4.15	<0.001	3.76	<0.001
hsa-miR-1909	hsa-miR-1909_st	-2.12	0.036	3.24	<0.001	3.79	<0.001
hsa-miR-1910	hsa-miR-1910_st	-2.30	0.004	2.50	0.039	3.80	0.002
hsa-miR-194-star	hsa-miR-194-star_st	-3.06	0.013				
hsa-miR-19a	hsa-miR-19a_st	-2.54	0.010			2.05	0.001
hsa-miR-19b	hsa-miR-19b_st	-2.36	0.006			3.10	0.001
hsa-miR-200a-star	hsa-miR-200a-star_st	-3.06	0.013			2.38	<0.001
hsa-miR-21	hsa-miR-21_st	2.08	0.001			2.03	0.008
hsa-miR-23a-star	hsa-miR-23a-star_st	-2.74	0.004	2.74	<0.001	3.85	<0.001
hsa-miR-25-star	hsa-miR-25-star_st	-3.55	0.001	3.27	<0.001	3.85	<0.001
hsa-miR-27a-star	hsa-miR-27a-star_st	-4.40	0.002	2.32	0.035	3.87	0.001
hsa-miR-3156-5p	hsa-miR-3156-5p_st	-2.65	0.005	2.24	0.021	3.88	0.001
hsa-miR-3175	hsa-miR-3175_st	-3.57	0.027	4.05	<0.001	3.90	<0.001
hsa-miR-3176	hsa-miR-3176_st	-3.01	0.003			2.23	0.003
hsa-miR-3178	hsa-miR-3178_st	-2.71	0.044	2.78	<0.001	3.91	<0.001
hsa-miR-3188	hsa-miR-3188_st	-3.02	0.011	2.37	0.018	3.95	<0.001
hsa-miR-3195	hsa-miR-3195_st	-2.87	0.019	2.95	<0.001	3.97	<0.001
hsa-miR-324-5p	hsa-miR-324-5p_st	-2.09	0.041				
hsa-miR-330-3p	hsa-miR-330-3p_st	-2.53	0.012	3.69	0.002	5.34	<0.001
hsa-miR-339-3p	hsa-miR-339-3p_st	-2.66	0.013			2.11	<0.001
hsa-miR-3613-3p	hsa-miR-3613-3p_st	-4.04	0.001	8.10	<0.001	3.98	<0.001
hsa-miR-3615	hsa-miR-3615_st	-2.21	0.002	3.20	0.001	3.98	<0.001
hsa-miR-362-5p	hsa-miR-362-5p_st	-3.99	0.016	2.68	0.005	4.00	<0.001
hsa-miR-3663-3p	hsa-miR-3663-3p_st	-2.32	0.013	2.68	<0.001	4.06	<0.001
hsa-miR-374b	hsa-miR-374b_st	2.34	0.029			3.79	0.001
hsa-miR-378	hsa-miR-378_st	-2.86	0.012			2.23	0.017
hsa-miR-378c	hsa-miR-378c_st	-3.33	0.014			3.17	0.009
hsa-miR-378d	hsa-miR-378d_st	-2.85	0.016			2.17	0.017
hsa-miR-378e	hsa-miR-378e_st	-2.22	0.008			2.15	0.008
hsa-miR-378f	hsa-miR-378f_st	-2.97	0.014			3.30	0.013
hsa-miR-378g	hsa-miR-378g_st	-2.59	0.020				
hsa-miR-378i	hsa-miR-378i_st	-2.91	0.013			2.36	0.013
hsa-miR-422a	hsa-miR-422a_st	-3.36	0.007			2.49	0.001
hsa-miR-423-3p	hsa-miR-423-3p_st	-2.11	0.036			2.09	0.002

hsa-miR-425-star	hsa-miR-425-star_st	-2.20	0.034			2.01	0.022
hsa-miR-4286	hsa-miR-4286_st	-3.51	0.003			2.37	0.001
hsa-miR-4322	hsa-miR-4322_st	-2.02	0.017	4.52	<0.001	4.08	<0.001
hsa-miR-4329	hsa-miR-4329_st	2.05	0.032	4.40	<0.001	4.09	<0.001
hsa-miR-4440	hsa-miR-4440_st	-2.68	0.023	2.74	0.010	4.13	<0.001
hsa-miR-4443	hsa-miR-4443_st	-3.07	0.031	2.35	0.001	4.17	<0.001
hsa-miR-4449	hsa-miR-4449_st	-3.83	0.005	2.82	0.005	4.18	<0.001
hsa-miR-4454	hsa-miR-4454_st	-3.29	0.004			2.07	0.001
hsa-miR-4492	hsa-miR-4492_st	-2.89	0.014	2.65	0.016	4.20	<0.001
hsa-miR-4505	hsa-miR-4505_st	-2.94	0.016	2.72	0.001	4.23	<0.001
hsa-miR-4507	hsa-miR-4507_st	-2.61	0.011	4.45	<0.001	4.27	<0.001
hsa-miR-4532	hsa-miR-4532_st	-2.69	0.035			2.18	0.001
hsa-miR-4634	hsa-miR-4634_st	-2.79	0.025	3.25	0.001	4.27	<0.001
hsa-miR-4651	hsa-miR-4651_st	-2.30	0.010	3.29	<0.001	4.31	<0.001
hsa-miR-4665-5p	hsa-miR-4665-5p_st	-2.04	0.021	3.20	0.022	4.34	0.004
hsa-miR-4668-5p	hsa-miR-4668-5p_st	-4.36	0.001			2.14	0.001
hsa-miR-4674	hsa-miR-4674_st	-2.75	0.010	2.60	0.037	4.34	0.001
hsa-miR-4690-5p	hsa-miR-4690-5p_st	-2.33	0.012	5.52	0.005	4.39	0.012
hsa-miR-4707-5p	hsa-miR-4707-5p_st	-2.47	0.021	2.69	0.004	4.40	<0.001
hsa-miR-4721	hsa-miR-4721_st	-2.39	0.005	8.73	<0.001	4.48	<0.001
hsa-miR-4734	hsa-miR-4734_st	-3.44	0.015	4.45	0.002	4.52	0.001
hsa-miR-4741	hsa-miR-4741_st	-2.37	0.020	3.12	0.008	4.53	0.001
hsa-miR-4745-5p	hsa-miR-4745-5p_st	-2.57	0.012	3.28	<0.001	4.58	<0.001
hsa-miR-4767	hsa-miR-4767_st	-2.13	0.005	3.24	<0.001	4.61	<0.001
hsa-miR-4786-5p	hsa-miR-4786-5p_st	-2.66	0.005			2.30	0.001
hsa-miR-4793-3p	hsa-miR-4793-3p_st	-2.57	0.043	2.88	0.008	4.63	<0.001
hsa-miR-500a	hsa-miR-500a_st	-2.39	0.030	5.66	<0.001	4.63	<0.001
hsa-miR-501-3p	hsa-miR-501-3p_st	-2.06	0.038			2.54	<0.001
hsa-miR-501-5p	hsa-miR-501-5p_st	-2.43	0.027	4.37	<0.001	4.66	<0.001
hsa-miR-513a-5p	hsa-miR-513a-5p_st	-2.09	0.015			2.32	0.001
hsa-miR-532-5p	hsa-miR-532-5p_st	-2.91	0.021	3.40	<0.001	4.66	<0.001
hsa-miR-652	hsa-miR-652_st	-2.42	0.048			2.03	<0.001
hsa-miR-663	hsa-miR-663_st	-2.59	0.048	6.45	0.001	4.69	0.004
hsa-miR-671-5p	hsa-miR-671-5p_st	-2.10	0.004				
hsa-miR-675	hsa-miR-675_st	-2.16	0.014	2.74	0.010	4.72	<0.001
hsa-miR-720	hsa-miR-720_st	-4.02	0.014			2.02	<0.001
hsa-miR-92b-star	hsa-miR-92b-star_st	-2.10	0.020	3.27	0.005	4.75	<0.001
hsa-miR-933	hsa-miR-933_st	-2.04	0.008			2.09	0.001
hsa-miR-939	hsa-miR-939_st	-2.12	0.035	6.62	<0.001	4.78	0.001
hsa-miR-93-star	hsa-miR-93-star_st	-3.24	0.008	3.80	0.001	4.81	<0.001
IL13RA2	ENSG00000123496_at	-2.13	0.012	4.43	0.004	7.42	<0.001
ITGA7	ENSG00000135424_at	2.18	0.018	9.81	<0.001	7.07	<0.001
LCN2	ENSG00000148346_at	-2.79	0.034	15.39	<0.001	9.02	<0.001
MAP1B	ENSG00000131711_at	2.13	0.004	4.32	<0.001	7.06	<0.001
mgh18S-121	mgh18S-121_st	-2.59	0.025			2.13	<0.001
mgh28S-2411	mgh28S-2411_st	-2.56	0.032	4.95	0.040	4.83	0.044

MGP	ENSG00000111341_at	2.69	0.012	5.52	<0.001	6.82	<0.001
MMP10	ENSG00000166670_at	-2.35	0.012	8.37	<0.001	8.18	<0.001
MMP3	ENSG00000149968_at	-3.78	0.017			2.56	0.009
MOCOS	ENSG00000075643_at	-3.10	0.001	6.66	<0.001	8.27	<0.001
MYH11	ENSG00000133392_at	2.69	0.013	2.06	0.026	2.13	0.006
MYL9	ENSG00000101335_at	2.17	0.008	6.35	<0.001	6.52	<0.001
OIP5	ENSG00000104147_at	-2.01	0.024	7.45	<0.001	8.84	<0.001
PBK	ENSG00000168078_at	-2.32	0.005	5.28	<0.001	8.68	<0.001
PCOLCE2	ENSG00000163710_at	2.51	0.007	3.85	<0.001	6.34	<0.001
PIGR	ENSG00000162896_at	-4.20	0.041			2.01	0.021
PRAP1	ENSG00000165828_at	2.15	0.032	4.11	0.015	6.05	<0.001
REG1A	ENSG00000115386_at	-9.45	0.016	4.69	0.018	14.47	<0.001
REG3A	ENSG00000172016_at	-8.46	0.006	10.84	<0.001	10.48	<0.001
SALL4	ENSG00000101115_at	2.02	0.005			2.46	0.012
SFRP4	ENSG00000106483_at	3.18	0.012			2.37	0.032
SLIT2	ENSG00000145147_at	2.11	0.003	4.15	<0.001	7.24	<0.001
SNORA24	ENSG00000207130_s_st	-3.22	0.010	2.01	0.009		
SNORA24	ENSG00000206903_s_st	-3.31	0.008			3.07	0.002
SNORA38	ENSG00000201042_s_st	-2.48	0.013	4.83	0.002	7.43	<0.001
SNORA38B	SNORA38B_s_st	-2.87	0.019			-2.26	0.022
SNORA38B	SNORA38B_s_st	-3.54	0.008			-2.35	0.001
SNORA38B	SNORA38B_s_st	-2.09	0.043	3.72	0.003	4.83	<0.001
SNORD116	ENSG00000202498_x_st	-2.18	0.006			2.37	<0.001
SPP1	ENSG00000118785_at	3.65	0.039			2.16	0.040
SRPX2	ENSG00000102359_at	2.66	0.017			2.12	0.028
TAGLN	ENSG00000149591_at	2.76	0.027	3.56	<0.001	6.04	<0.001
THBS4	ENSG00000113296_at	2.66	0.021			-3.40	0.003
TYMS	ENSG00000176890_at	-2.11	0.003	8.91	<0.001	7.73	<0.001
U104	U104_st	-2.22	0.005			-4.85	0.023
U105B	U105B_st	-2.30	0.018			-3.61	<0.001
U23	U23_st	-2.89	0.001	2.76	0.004	4.88	<0.001
U23	U23_st	-2.91	0.010	3.13	0.003	5.08	<0.001
U28	U28_st	-2.62	0.009	4.18	<0.001	5.10	<0.001
U3	U3_s_st	-2.44	0.010	3.57	0.030	5.14	0.004
U31	U31_st	-3.10	0.014	3.40	0.001	5.21	<0.001
U3-2	U3-2_s_st	-2.19	0.020	4.52	0.029	5.25	0.014
U3-3	U3-3_s_st	-2.29	0.016	3.70	0.008	5.25	0.001
U34	U34_st	-2.30	0.024			2.08	<0.001
U3-4	U3-4_s_st	-2.65	0.009	3.05	0.026	5.27	0.001
U36B	U36B_st	-2.03	0.023	3.53	0.005	5.33	<0.001
U37	U37_st	-2.34	0.016	3.03	<0.001	5.37	<0.001
U38A	U38A_x_st	-3.03	0.012	-3.28	0.004	-3.75	<0.001
U38A	U38A_x_st	-3.16	0.014	3.09	0.019	5.39	0.001
U43	U43_st	-2.10	0.043	3.32	0.001	5.41	<0.001
U43	U43_st	-2.16	0.023	3.57	0.006	5.43	<0.001
U48	U48_st	-2.79	0.019	3.63	<0.001	5.44	<0.001

U50B	U50B_st	-2.28	0.026	-3.15	0.001		
U58C	U58C_x_st	-2.26	0.008				
U68	U68_st	-2.07	0.043	7.53	<0.001	5.50	<0.001
U68	U68_st	-2.37	0.014	4.34	0.006	5.50	0.001
U74	U74_x_st	-2.49	0.018	4.12	<0.001	5.56	<0.001
U75	U75_x_st	-2.00	0.023	3.38	0.005	5.68	<0.001
U75	U75_x_st	-2.46	0.038	2.91	0.034	5.71	0.001
U99	U99_st	-2.22	0.005	8.14	<0.001	5.76	<0.001
ZBTB20	ENSG00000181722_at	2.00	0.001			2.79	<0.001
ZEB1	ENSG00000148516_at	2.00	0.008	8.27	<0.001	7.24	<0.001
ZG16B	ENSG00000162078_at	-2.39	0.040			2.22	0.006

Only genes with an FDR<.05 for at least one comparisons are shown; MTX: metastatic colorectal cancer; non-MTX: non-metastatic colorectal cancer; hsa-miR: human micro-RNA. FDR: false discovery rate.

Supplementary table 2. Commercial antibodies used in the study.

Antibody	Clone	Dilution	Manufacturer	Interpretation	Positive control
CK7	RN7	1:100	Novocastra	Cytoplasmic stain	Bladder (epithelium)
CK20	K_s20.8	Prediluted	Novocastra	Cytoplasmic stain	Colon (epithelium)
CDX2	CDX2-88	Prediluted	Biocare	Nuclear stain	Colon (epithelium)
MLH1<br>MSH2<br>MSH6<br>PMS2	G168-15<br>FE11<br>BC/44<br>M0R4G	1:25<br>Prediluted<br>Prediluted<br>1:50	BD Pharmingen<br>Biocare<br>Biocare<br>Novocastra	Negative: no tumor nuclei stained with internal positive control<br>Positive: at least one tumor nucleus stained with positive internal control	Tonsil
c-Myc	Y69	Prediluted	Master Diagnostica	Negative: <10% positive nuclei<br>Positive: ≥10% positive nuclei	Tonsil
Her2	Bond Oracle HER2 IHC System		Leica	0+: No reactivity or no membranous reactivity in any tumor cell<br>1+: Tumor cell cluster with a faint/barely perceptible membranous reactivity irrespective of percentage of tumor cells stained<br>2+: Tumor cell cluster with a weak to moderate complete, basolateral, or lateral membranous reactivity, irrespective of percentage of tumor cells stained<br>3+: Tumor cell cluster with a strong complete, basolateral, or lateral membranous reactivity, irrespective of percentage of tumor cells stained	Her2 control slide (Bond Oracle)
p53	DO-7	1:100	Novocastra	0: no nuclei stained<br>1: 0-10% positive nuclei<br>2: ≥10% positive nuclei	Tonsil
β-catenin	Polyclonal	Prediluted	Master Diagnostica	Negative: <30% positive nuclei<br>Positive: ≥30% positive nuclei	Colon (epithelium)
CEA	12-140-1	1:100	Novocastra	Negative: no stained cells or non-specific stain<br>Positive: any specific cytoplasmic stain	Colon (adenocarcinoma)
EGFR	2-18C9	Prediluted	DAKO	0: no membranous stain in any tumor cell<br>1-10%: percentage of tumor cells with membranous stain<br>10-35%: percentage of tumor cells with membranous stain<br>>35%: percentage of tumor cells with membranous stain	Placenta
Ki-67	SP6	1:100	Master Diagnostica	Negative: <50% positive nuclei<br>Positive: ≥50% positive nuclei	Tonsil
